# Subgraph Neural Networks Enhanced by Global Similarity for Drug Repositioning

**DOI:** 10.1007/s12539-025-00747-x

**Published:** 2025-10-29

**Authors:** Chengyan Zhou, Xinliang Sun, Xiang Du, Min Zeng, Min Li

**Affiliations:** 1 School of Software, Xinjiang University, Urumqi, 830091 China; 2 School of Computer Science and Engineering, Central South University, Changsha, 410083 China; 3 School of Information Engineering, Jiangxi University of Science and Technology, Ganzhou, 341000 China

**Keywords:** Drug repositioning, Graph convolutional network, Graph representation, Global similarity

## Abstract

**Abstract:**

Drug repositioning is a promising strategy for accelerating drug development and reducing costs by identifying potential indications for existing drugs. Recently, technological advancements have enabled the development of numerous graph convolutional network (GCN)-based methods for drug repositioning. However, many existing methods overlook the distinct roles of nodes within drug-disease association graphs, limiting their ability to learn effective representations. To address this limitation, we propose a subgraph neural network enhanced by global similarity for drug repositioning, termed GSESNN. Specifically, GSESNN first extracts the subgraph of each drug-disease pair from the entire drug-disease graph. Then, GCN and a sort pooling strategy are utilized to learn the subgraph representation. In addition, to distinguish between different drug-disease pairs with the identical subgraph topology, GSESNN utilizes GCN to learn the similarity information of drugs and diseases, fusing it with the subgraph representation to produce the final representation. Finally, we regard the drug-disease association prediction as a graph classification task. Experimental results show that GSESNN outperforms the baseline model in drug repositioning tasks. Case studies on Alzheimer’s disease and Gastric Cancer further demonstrate that our model successfully identifies more accurate drug-disease associations, highlighting its potential for practical applications in drug discovery.

**Graphical Abstract:**

**Figure:**
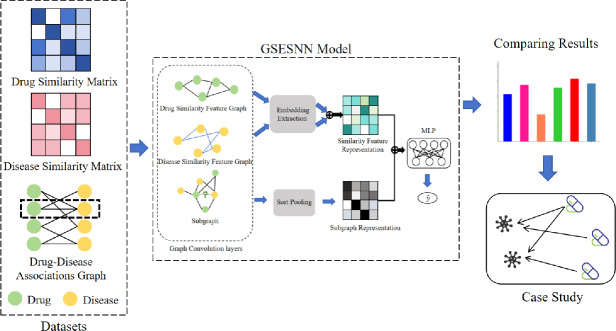

## Introduction

Drug repositioning, also known as drug repurposing, is a strategy aimed at exploring existing drugs to identify new therapeutic opportunities [1]. This approach leverages established clinical data and the known safety profiles of drugs, significantly reducing the risks associated with research and development while increasing the likelihood of success. Studies indicate that the cost of drug repositioning is approximately $300 million, compared with the $2 to $3 billion typically required for developing a novel chemical entity [2]. Notably, multiple drugs have been successfully repositioned to treat novel indications. A classic example is Aspirin, which was originally developed as a painkiller but has since been repositioned for the treatment and prevention of cardiovascular disease and colorectal cancer [3, 4].

In recent years, computing-based methods have received significant attention due to their effectiveness in drug repositioning tasks, delivering high-quality prediction results. These approaches are primarily data-driven, extracting latent features from known drug-disease interaction data and employing various machine learning techniques to identify potential new indications for existing drugs. Broadly, these methods can be divided into three categories: (1) matrix factorization-based and matrix completion-based methods; (2) feature-based methods; (3) deep learning-based methods [5]. Matrix factorization-based methods aim to map the drug-disease association matrix to a low-rank feature space by minimizing the reconstruction error of latent embeddings. The similarity matrices of drugs and diseases serve as additional constraints, incorporating biological side information. This approach is widely used in drug repositioning due to its flexibility in integrating prior knowledge and remains a powerful tool with promising applications. For example, Xuan et al. [6] develops DivePred, a method based on non-negative matrix factorization, to predict potential drug-disease associations. DivePred integrates various drug similarities, reflecting features from different perspectives. Moreover, MLMC [7] introduces an integrated matrix completion model that incorporates multi-source biological data (such as gene expression, protein interaction, etc.) to comprehensively capture the complex relationships between drugs and diseases. Feature-based learning methods extract features from drugs, diseases, and biological networks and preprocess them, then predict drug-disease associations based on these processed features. For example, LBMFF [8] effectively integrates known drugs, diseases, side effect, and target associations, along with literature-derived semantic features from public databases. A GCN with an attention mechanism is also employed to capture structural information from the integrated similarity matrix and known drug-disease associations. In addition, deep learning-based methods are increasingly employed for drug repositioning. For example, KGCNH [9] employs a relation-aware attention mechanism to calculate attention scores for entities in the knowledge graph under different relations, integrated with a heuristic search module to explore more optimal drug and disease embeddings. Through neighborhood aggregation, the model derives the final embeddings of drugs and diseases, thus enabling accurate prediction of drug–disease associations. Each of these three methods has its own advantages. Matrix factorization-based and matrix completion-based methods can effectively predict unknown drug-disease associations with sparse data, while feature-based methods utilize cross-modal complementary information obtained by integrating multiple biometric information to improve prediction accuracy. Finally, deep learning-based methods are able to capture complex, nonlinear, and high-order relationships between drugs and diseases, thus discovering complex patterns that may be missed by traditional models.

Although these methods perform well in drug repositioning, most of them rely on unique features or structures of a particular network, thus limiting their ability to adapt to different networks. Moreover, each drug-disease association network contains a large number of drug nodes and disease nodes. These nodes are connected through multiple links, and each node plays a unique role in different links. However, many existing methods overlook the distinct roles of nodes in drug-disease association graphs, limiting their ability to learn effective representations.

To address the above problem, we propose a GCN-based drug repositioning method that fuses subgraph representation with global similarity feature representation. To implement this method, we firstly extract the subgraph of each drug-disease pair from the entire drug-disease graph, and learn its subgraph representation utilizing GCN. Secondly, we utilize the similarity matrices of drugs and diseases to construct *k*-nearest neighbors (*k*NN) graphs as their similarity feature graphs. We then utilize GCN to learn global similarity feature representations from these similarity feature graphs. To distinguish between identical subgraph topological information for different drug-disease pairs, we fuse subgraph representation and global similarity feature representation to produce the final representation, and use a weight factor to balance their relative contributions. This ensures that the model captures and integrates important information from both types of representations, thereby enhancing model performance.

In summary, the main contributions of this work are as follows:

- We employ GCN to learn subgraph representation and global similarity feature representation from subgraphs and similarity feature graphs.
- Considering that identical subgraph topology may be shared by different node pairs in the association graph, we fuse the global similarity feature representation with the subgraph representation to distinguish the representations of different node pairs. In addition, we employ a weight factor to balance the contributions of two representations in the model.
- Experimental results on the benchmark dataset demonstrate that GSESNN outperforms the baseline model in terms of AUROC and AUPRC. The case studies further demonstrate the potential of GSESNN in practical applications.

## Materials and Methods

**Fig. 1:**
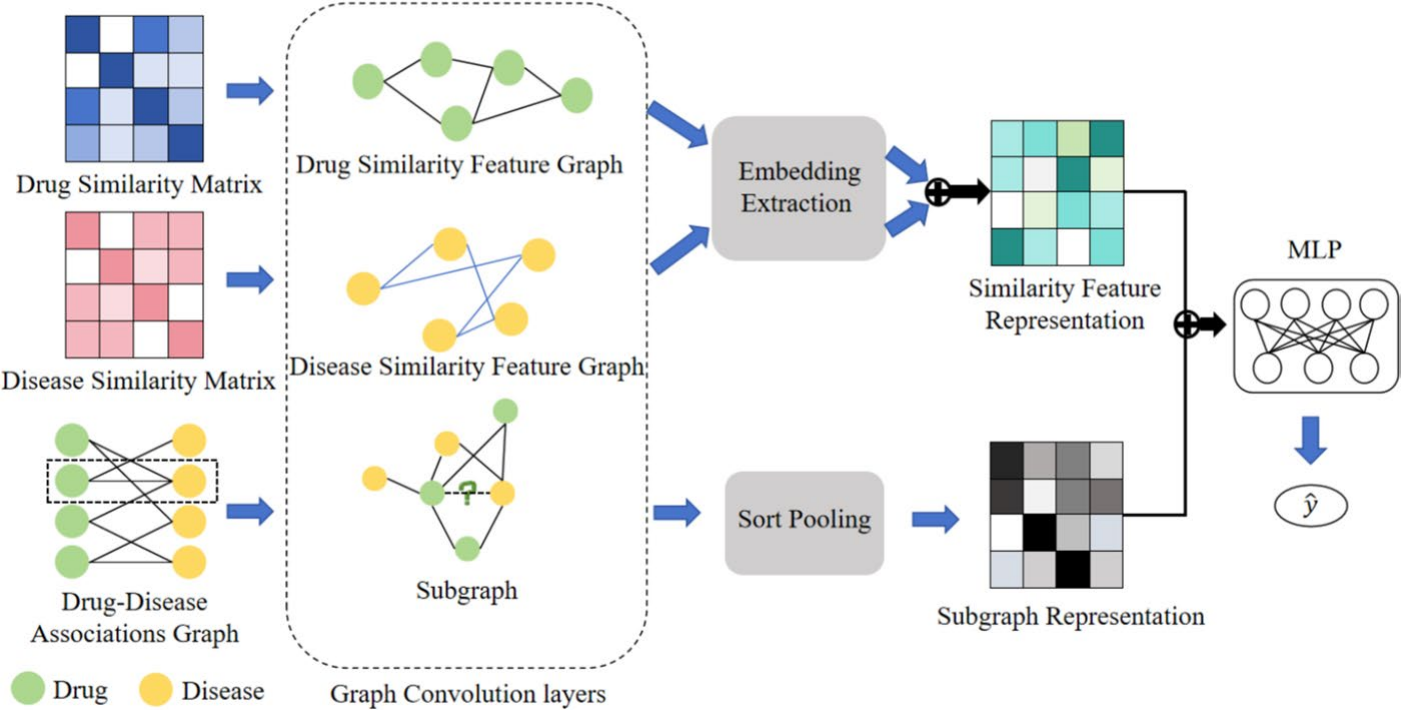
The framework of GSESNN. **a** GSESNN extracts a subgraph for each drug-disease pair. By employing GCN and sort pooling, GSESNN learns the subgraph representation. **b** GSESNN employs GCN to learn global similarity feature representations from the similarity feature graph, and fuses them into the corresponding subgraph representations of drug-disease pairs. **c** GSESNN predicts potential drug-disease associations as binary classification of the graphs after fusing subgraph representation and global similarity feature representation

In this section, we preliminarily describe the benchmark datasets used in the proposed model. We then introduce the GSESNN model framework, which mainly consists of three parts, as illustrated in Fig. 1: (1) extracting subgraphs for drug-disease pairs and utilizing GCN to learn subgraph representation; (2) learning global similarity feature representations from the similarity feature graph, and fusing them into the corresponding subgraph representations of drug-disease pairs; (3) predicting drug-disease associations after fusing the subgraph representation and the global similarity feature representation.

### Dataset

To comprehensively evaluate the performance of the proposed model, we use three publicly available benchmark datasets: Gdataset [10], Cdataset [11], and LRSSL [12], which are widely used in drug repositioning tasks. Table 1 summarizes detailed information about these datasets. Gdataset, considered as the gold standard dataset, contains 1933 verified associations between drugs and diseases. These associations originate from 593 drugs in the DrugBank [13] and 313 diseases listed in the OMIM database [14]. Cdataset consists of 663 drugs, 409 diseases, and 2532 known drug-disease associations. The final dataset, LRSSL, comprises 3051 verified drug-disease associations involving 763 drugs and 681 diseases. Moreover, to construct similarity feature graphs for drugs and diseases, we utilize similarity feature information of drugs and diseases in the datasets. In the datasets, drug-drug similarity information is defined by Tanimoto scores of 2D molecular fingerprints between drugs, while disease-disease similarity information is calculated by text mining that analyzes semantic information between diseases.

**Table 1:** Statistics of the three benchmark datasets

Dataset	Drugs	Diseases	Known associations	Sparsity
Gdataset	593	313	1933	0.0104
Cdataset	663	409	2532	0.0093
LRSSL	763	681	3051	0.0059

### Subgraph Extraction

**Fig. 2:**
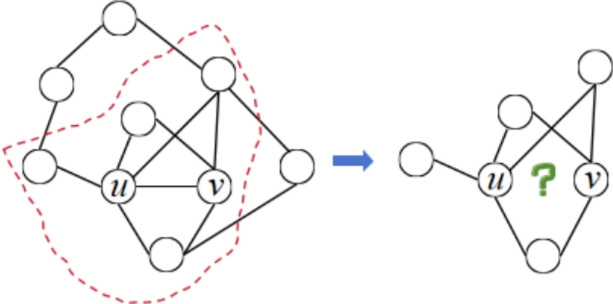
An illustration of the subgraph extraction

Based on the known drug-disease association relationships presented in the datasets, we construct a drug-disease associations graph. We regard all known drug-disease associations as positive samples and randomly select an equal number of negative samples from unknown associations. For each drug-disease node pair, we take these nodes as centers and extract their *h*-hop neighbors to construct a subgraph. To prevent information leakage from positive samples, we remove the target drug-disease associations from the subgraph during this process. As illustrated in Fig. 2, for the node pair (*u*, *v*), we extract their 1-hop neighbors to construct a subgraph.

### Subgraph Representation Learning

After extracting the subgraphs for the target drug-disease node pairs, we utilize a graph-level GCN model to learn the subgraph representation. The graph-level GCN consists of two main components: (1) message propagation layers that capture neighbor information to produce node representations; (2) a sort pooling layer that aggregates these node embeddings into a comprehensive graph-level representation.

To aggregate the neighbor information of the central node within the subgraph, a GCN [15] is utilized to learn the node representations. Moreover, to fully acquire the structural information of the subgraphs, we stack *L* layers of message passing layers. The convolution operation for the subgraph $P_{ij}$ in the *l*th layer is defined as follows:

1 $$\begin{aligned} {\boldsymbol{{Z}}}^{(l)} = \mathrm{ReLU}({\widetilde{{\boldsymbol{{D}}}}^{-\frac{1}{2}}}{\boldsymbol{\widetilde{{A}}}^\mathrm{p}}{\widetilde{{\boldsymbol{{D}}}}^{-\frac{1}{2}}}{\boldsymbol{{Z}}}^{(l-1)}{\boldsymbol{{W}}}^{(l)}) \end{aligned}$$

where ${\boldsymbol{{Z}}}^{(l)} \in {{\textbf {R}}}^{N \times d_l}$ represents the output node embeddings at layer *l*, *N* is the number of nodes in the subgraph, and $d_l$ is the number of output channels at that layer. For *l* = 1, the initial node features ${\boldsymbol{{Z}}}^0$ = ***X*** is a one-hot encoding of the node information within the subgraph. ${\boldsymbol{{A}}}^\mathrm{p}$ is the adjacency matrix of the subgraph $P_{ij}$. The adjacency matrix ${\boldsymbol{\widetilde{{A}}}^\mathrm{p}} = {\boldsymbol{{A}}}^\mathrm{p} + {\boldsymbol{{I}}}$ corresponds to the subgraph $P_{ij}$ with added self-connections, where ***I*** is the identity matrix. $\widetilde{{\boldsymbol{{D}}}}$ is a diagonal degree matrix with $\widetilde{D}_{ii} = {\sum _{j}}\widetilde{A}^\mathrm{p}_{ij}$, and ${\boldsymbol{{W}}}^{(l)}$ represents the trainable parameter matrix at layer *l*. $\mathrm{ReLU}(\cdot )$ is a nonlinear activation function. After learning the representations from the *L* layers, the final node representation ${\boldsymbol{{Z}}}^{1:L}$ is then defined as follows:

2 $$\begin{aligned} {\boldsymbol{{Z}}}^{1:L} = \mathrm{concat}({\boldsymbol{{Z}}}^{(1)}, {\boldsymbol{{Z}}}^{(2)}, . .., {\boldsymbol{{Z}}}^{(L)}) \end{aligned}$$

where ${\boldsymbol{{Z}}}^{1:L} \in {{\textbf {R}}}^{N \times {\sum _{1}^{L}}d_l }$ is the concatenated output of *L* graph convolutional layers, and each row represents a node feature embedding and each column represents a feature channel.

To obtain the final node representations, these embeddings are integrated into the graph feature vector through a sort pooling layer [16]. Specifically, since ${\boldsymbol{{Z}}}^{(L)}$ aggregates the most comprehensive neighbor information, we first sort nodes in descending order based on the value in its last channel. If two nodes have the identical value in the last channel, the tie is broken by comparing their values in the second to last channel, and so on. After sorting the node features in a consistent order, the size of the number of nodes is uniformly fixed to *K*. If N>K, the output node representation is truncated by deleting the last N-K rows to make it of length *K*. If N<K, the output is extended by adding K-N zero rows. The output of sort pooling layer, denoted as ${\boldsymbol{{Z}}}_\mathrm{sp}$, is a $K \times {\sum _{1}^{L}} d_l$ tensor. Next, the reshaping operation is used to map ${\boldsymbol{{Z}}}_\mathrm{sp}$ into a $1 \times K ({\sum _{1}^{L}} d_l )$ vector. To sequentially apply filters on ${\boldsymbol{{Z}}}_\mathrm{sp}$, we employ a one-dimensional convolutional layer with filter size and step ${\sum _{1}^{L}} d_l$. After that, a max-pooling layer and one-dimensional convolutional layer are utilized to capture local subgraph patterns on the node sequences in ${\boldsymbol{{Z}}}_\mathrm{sp}$, and the output feature vector is denoted as ${\boldsymbol{{Z}}}_\mathrm{p}$. In the final step, fully connected layers classify each subgraph $P_{ij}$ with ${\boldsymbol{{Z}}}_\mathrm{p}$ as follows:

3 $$\begin{aligned} {\boldsymbol{{Z}}}_\mathrm{S} = {\boldsymbol{{W}}}_2 \cdot \mathrm{ReLU}({{\boldsymbol{{W}}}_1}{{\boldsymbol{{Z}}}_\mathrm{p}} + {\boldsymbol{{b}}}_1) + {\boldsymbol{{b}}}_2 \end{aligned}$$

where ${\boldsymbol{{Z}}}_\mathrm{S}$ represents subgraph learning representation. The variables ${\boldsymbol{{W}}}_1$ and ${\boldsymbol{{W}}}_2$ denote trainable weights, while ${\boldsymbol{{b}}}_1$ and ${\boldsymbol{{b}}}_2$ represent the corresponding bias terms.

### Global Similarity Feature Representation Learning

Each drug and disease has distinct global similarity feature representation, so we utilize their global similarity feature representation to distinguish subgraph representation of the node pairs with identical subgraph. To enhance the global similarity features of drugs and diseases in the feature space, we construct their *k*NN graphs as similarity feature graphs based on their respective similarity matrices. Here, we denote the drug similarity matrix by ${\boldsymbol{{X}}}^\mathrm{r} \in {{\textbf {R}}}^{n \times n}$, where *n* is the number of drugs. The adjacency matrix of drug *k*NN graph is represented by the binary matrix ${\boldsymbol{{A}}}^\mathrm{r} \in {{\textbf {R}}}^{n \times n}$, where each entry of ${\boldsymbol{{A}}}^\mathrm{r}$ is constructed based on the similarity of each pair of drugs. The entry $A_{ij}^\mathrm{r}$ of matrix ${\boldsymbol{{A}}}^\mathrm{r}$ is defined as

4 $$\begin{aligned} A_{ij}^\mathrm{r} = {\left\{ \begin{array}{ll} 1, & \quad \text {if } r_j \in \widetilde{N}_k(r_i) \\ 0, & \quad \text {otherwise} \end{array}\right. } \end{aligned}$$

where $\widetilde{N}_k(r_i) = \{r_i\} \cup N_k(r_i)$ is a set of $r_i$’s extended *k*-nearest neighbors including $r_i$, and $N_k(r_i)$ is the *k*-nearest neighbors of drug $r_i$. In the same way, we denote the disease similarity matrix by ${\boldsymbol{{X}}}^\mathrm{d} \in {{\textbf {R}}}^{m \times m}$, where *m* is the number of diseases. The adjacency matrix of disease *k*NN graph is represented by the binary matrix ${\boldsymbol{{A}}}^\mathrm{d} \in {{\textbf {R}}}^{m \times m}$, where each entry of ${\boldsymbol{{A}}}^\mathrm{d}$ is constructed based on the similarity of each pair of diseases. The entry $A_{ij}^\mathrm{d}$ of matrix ${\boldsymbol{{A}}}^\mathrm{d}$ is defined as

5 $$\begin{aligned} A_{ij}^\mathrm{d} = {\left\{ \begin{array}{ll} 1, & \quad \text {if } d_j \in \widetilde{N}_k(d_i) \\ 0, & \quad \text {otherwise} \end{array}\right. } \end{aligned}$$

where $\widetilde{N}_k(d_i) = \{d_i\} \cup N_k(d_i)$ is a set of $d_i$’s extended *k*-nearest neighbors including $d_i$, and $N_k(d_i)$ is the *k*-nearest neighbors of disease $d_i$.

With the drug *k*NN graph $G_\mathrm{r}=({\boldsymbol{{A}}}^\mathrm{r}, {\boldsymbol{{X}}}^\mathrm{r})$ and the disease *k*NN graph $G_\mathrm{d}=({\boldsymbol{{A}}}^\mathrm{d}, {\boldsymbol{{X}}}^\mathrm{d})$ in feature space, we employ the GCN [15] to represent the *l*-layer output of the constructed feature graphs:

6 $$\begin{aligned} {\left\{ \begin{array}{ll} {\boldsymbol{{Z}}}_\mathrm{r}^{(l)} = \mathrm{ReLU}({\widetilde{{\boldsymbol{{D}}}}_\mathrm{r}^{-\frac{1}{2}}}{\boldsymbol{\widetilde{{A}}}^\mathrm{r}}{\widetilde{{\boldsymbol{{D}}}}_\mathrm{r}^{-\frac{1}{2}}}{\boldsymbol{{Z}}}_\mathrm{r}^{(l-1)}{\boldsymbol{{W}}}_\mathrm{r}^{(l)}) \\ {\boldsymbol{{Z}}}_\mathrm{d}^{(l)} = \mathrm{ReLU}({\widetilde{{\boldsymbol{{D}}}}_\mathrm{d}^{-\frac{1}{2}}}{\boldsymbol{\widetilde{{A}}}^\mathrm{d}}{\widetilde{{\boldsymbol{{D}}}}_\mathrm{d}^{-\frac{1}{2}}}{\boldsymbol{{Z}}}_\mathrm{d}^{(l-1)}{\boldsymbol{{W}}}_\mathrm{d}^{(l)}) \end{array}\right. } \end{aligned}$$

where ${\boldsymbol{{Z}}}_\mathrm{r}^{(l)} \in {{\textbf {R}}}^{n \times d_l}, {\boldsymbol{{Z}}}_\mathrm{d}^{(l)} \in {{\textbf {R}}}^{m \times d_l}$ represent the information propagated at the *l*-th layer for drugs and diseases, respectively. $d_l$ is the number of output channels at the *l*-th layer. ${\boldsymbol{{W}}}_\mathrm{r}^{(l)}, {\boldsymbol{{W}}}_\mathrm{d}^{(l)}$ are the weight matrices of the *l*-th layer in GCN. The initial similarity features ${\boldsymbol{{Z}}}_\mathrm{r}^{(0)} = {\boldsymbol{{X}}}^\mathrm{r}, {\boldsymbol{{Z}}}_\mathrm{d}^{(0)} = {\boldsymbol{{X}}}^\mathrm{d}$; $\widetilde{{\boldsymbol{{D}}}}_\mathrm{r}, \widetilde{{\boldsymbol{{D}}}}_\mathrm{d}$ represent the diagonal degree matrix of ${{\boldsymbol{\widetilde{A}}}^\mathrm{r}}$ and ${{\boldsymbol{\widetilde{A}}}^\mathrm{d}}$, respectively. The adjacency matrix ${{\boldsymbol{\widetilde{A}}}^\mathrm{r}} = {\boldsymbol{{A}}}^\mathrm{r} + {\boldsymbol{{I}}}$ corresponds to the drug *k*NN graph $G_\mathrm{r}$ with added self-connections, where ***I*** is the identity matrix. In the same way, the adjacency matrix ${{\boldsymbol{\widetilde{A}}}^\mathrm{d}} = {\boldsymbol{{A}}}^\mathrm{d} + {\boldsymbol{{I}}}$ corresponds to the disease *k*NN graph $G_\mathrm{d}$ with added self-connections. $\mathrm{ReLU}(\cdot )$ is a nonlinear activation function.

To distinguish the identical subgraph topological information of different drug-disease pairs, we extract the corresponding global similarity feature representation based on the target drug-disease node pairs of the extracted subgraph. For each subgraph $P_{ij}$, we respectively extract the corresponding output embeddings ${\boldsymbol{{Z}}}_i$ and ${\boldsymbol{{Z}}}_j$ of the target drug *i* and disease *j* from the output embeddings of the last layer of the drugs and the diseases. We concatenate the embeddings of the two nodes to obtain the final global similarity feature representation. The final global similarity feature representation ${\boldsymbol{{Z}}}_\mathrm{F}$ is then defined as follows:

7 $$\begin{aligned} {\boldsymbol{{Z}}}_\mathrm{F} = [{\boldsymbol{{Z}}}_i, {\boldsymbol{{Z}}}_j] \end{aligned}$$

We then perform a linear weight fusion of the two learned representations and set a weight factor for the subgraph representation. The sum of the weight factors of the two representations is 1. By employing weight fusion, we produce a distinct final representation for each drug-disease pair as follows:

8 $$\begin{aligned} {\boldsymbol{{Z}}} = \lambda {{\boldsymbol{{Z}}}_\mathrm{S}} + (1 - \lambda ){{\boldsymbol{{Z}}}_\mathrm{F}} \end{aligned}$$

where ${\boldsymbol{{Z}}}$, ${{\boldsymbol{{Z}}}_\mathrm{S}}$, ${{\boldsymbol{{Z}}}_\mathrm{F}}$ denote the final representation, subgraph representation and global similarity feature representation, respectively. λ denotes the weight factor. Finally, the fully connected layers perform classification for the final representation of each drug-disease node pair as follows:

9 $$\begin{aligned} \hat{y} = {\boldsymbol{{W}}}_2 \cdot \mathrm{ReLU}({{\boldsymbol{{W}}}_1}{{\boldsymbol{{Z}}}} + {\boldsymbol{{b}}}_1) + {\boldsymbol{{b}}}_2 \end{aligned}$$

where $\hat{y}$ represents the probability of the association between drug *i* and disease *j*. The variables ${\boldsymbol{{W}}}_1$ and ${\boldsymbol{{W}}}_2$ denote trainable weights, while ${\boldsymbol{{b}}}_1$ and ${\boldsymbol{{b}}}_2$ represent the corresponding bias terms.

### Model Training

We transform the drug-disease association prediction problem to the graph classification task, and apply the cross-entropy loss function to train the model. Known drug-disease associations in the dataset are considered as positive samples, and unknown associations are considered as negative samples. The cross-entropy loss function is defined as follows:

10 $$\begin{aligned} L = -\sum _{(i,j)} y_{ij} \cdot \mathrm{log}(\hat{y}_{ij}) \;+ (1-y_{ij}) \cdot \mathrm{log}(1\;-\hat{y}_{ij}) \end{aligned}$$

where (*i*, *j*) represents a pair of drug *i* and disease *j*. $y_{ij}$ represents the truth-value label, and $y_{ij} \in \{0,1\}$. $\hat{y}_{ij}$ is the predicted probability of association between of drug *i* and disease *j*. To optimize the model, we employ the Adam optimizer. We train the model in a denoising setup by randomly dropping out all outgoing messages of a particular edge with a fixed probability. We also employ standard dropout [17] to the prediction layer.

## Results

### Parameter Setting

In GSESNN, various hyperparameters influence model performance, including the total training epochs α, the learning rate $\eta \in \{0.0001, 0.001, 0.01, 0.1\}$, the node dropout rate $\gamma _1$, the edge dropout rate $\gamma _2$, the feature embeddings dropout rate $\gamma _3$, the hop depth of subgraph extraction $\textit{h} \in \{1, 2, 3\}$, the weight factor for the contribution of subgraph representation $\lambda \in \{0.1, 0.2,..., 0.9\}$, and the number of neighbors in the similarity feature graph *K*. After empirical tuning, we set these values as follows: α = 50, η = 0.0001, $\gamma _1$ = 0.4, $\gamma _2$ = 0.1, $\gamma _3$ = 0.1, *h* = 2, λ = 0.7 and *K* = 2. For parameters, i.e. *h* and λ, the detailed tuning process is described in Sect. 3.5. For fair comparison, we use the default parameter settings specified in the respective original papers for the competing models.

### Baseline Model

To assess the effectiveness of our proposed model, we compare GSESNN with five leading drug repositioning methods. The baseline methods are described below:

- SCMFDD [18] is a matrix factorization method, which maps the high dimensional drug and disease latent vectors to a low dimensional space for prediction, and incorporates drug and disease similarities as constraints.
- iDrug [19] is a cross-network framework. It integrates drug repositioning and drug-target prediction into a unified network, which utilizes drugs as a bridge.
- DRWBNCF [20] models the complex drug–disease associations based on the weighted bilinear neural collaborative filtering approach.
- NIMCGCN [21] is a GCN-based method for miRNA-disease association prediction, which is demostrated to have great potential for drug repositioning.
- WIGRL [22] designs specific encoders for drug similarity networks, disease similarity networks and drug-disease association networks, respectively. By weighted fusion of information from different networks, more representative unified vectors are obtained to enhance the model’s representation of drug-disease associations.

### Performance of GSESNN in the Cross-validation

We perform a 10-fold cross-validation to rigorously evaluate the performance of our method. All known drug-disease associations are regarded as positive samples and randomly divided into 10 equal subsets. In each fold, nine subsets are used as the positive training sets, while the remaining subset serves as the positive testing set. To balance the data, we randomly select an equal number of negative samples for both training and testing sets. For performance evaluation, we employ the area under the receiver operating characteristic curve (AUROC) and the area under the precision-recall curve (AUPRC), which are widely recognized in drug repositioning prediction tasks. To ensure robust and reliable performance estimates, we repeat the 10-fold cross-validation procedure ten times, reporting the average results. Table 2 summarizes the results from three benchmark datasets.

**Table 2:** Performance comparison of 10-fold cross validation prediction results between our method and baselines over Gdataset, Cdataset and LRSSL datasets

Metric	Dataset	SCMFDD	iDrug	DRWBNCF	NIMCGCN	WIGRL	GSESNN
AUROC	Gdataset	0.7731 ± 0.0196	0.9078 ± 0.0158	0.9061 ± 0.0149	0.8234 ± 0.0157	0.9131 ± 0.0103	**0**.**9454** ± **0**.**0118**
	Cdataset	0.7896 ± 0.0157	0.9294 ± 0.0137	0.9277 ± 0.0157	0.8393 ± 0.0087	0.9247 ± 0.0127	**0**.**9575** ± **0**.**0087**
	LRSSL	0.7698 ± 0.0211	0.8993 ± 0.0104	0.9232 ± 0.0128	0.7581 ± 0.0123	0.9104 ± 0.0137	**0**.**9520** ± **0**.**0082**
AUPRC	Gdataset	0.7749 ± 0.0210	0.9265 ± 0.0127	0.9307 ± 0.0109	0.8590 ± 0.0156	0.9193 ± 0.0126	**0**.**9527** ± **0**.**0106**
	Cdataset	0.7878 ± 0.0180	0.9454 ± 0.0098	0.9476 ± 0.0099	0.8728 ± 0.0092	0.9291 ± 0.0065	**0**.**9622** ± **0**.**0078**
	LRSSL	0.7860 ± 0.0203	0.9212 ± 0.0080	0.9339 ± 0.0100	0.7962 ± 0.0096	0.9163 ± 0.0113	**0**.**9580** ± **0**.**0071**

The best reported result are bolded

Our findings indicate that GSESNN consistently outperforms other methods in AUROC and AUPRC metrics across the three datasets. On Gdataset, GSESNN achieves an AUROC of 0.9454, marking an improvement of 3.23 percentage points over the second-best approach, WIGRL. For Cdataset and LRSSL, GSESNN attains AUROC values of 0.9575 and 0.9520, outperforming iDrug and DRWBNCF by 2.81 percentage points and 2.88 percentage points, respectively. In terms of AUPRC, GSESNN also achieves superior performance, showing an average improvement of 2.20 percentage points over DRWBNCF on Gdataset, and enhancements of approximately 1.46 percentage points and 2.41 percentage points on Cdataset and LRSSL datasets, respectively. These results highlight the superiority of our proposed method for drug repositioning tasks.

The baseline approach achieves the results in Table 2 by incorporating additional information. For example, iDrug utilizes drug-target interaction information as a cross-domain bridge. Although our model also uses additional information, our model utilizes the more important drug similarity and disease similarity information. The results in the table demonstrate the superiority of our approach. In addition, comparing the two models NIMCGCN and DRWBNCF, the results of GSESNN on the three datasets also far exceed them, which proves the importance of utilizing target drug-disease pairs for feature representation learning.

### Predicting Indications for New Drugs

**Fig. 3:**
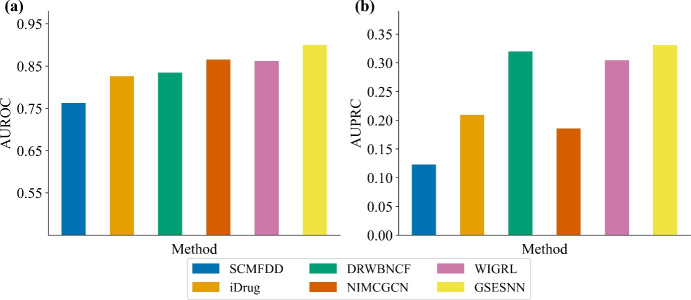
Performance of all methods in predicting potential indications for new drugs on Gdataset

The newly predicted drug-disease associations provide valuable insights for drug repositioning tasks. Therefore, we conduct a new experiment to evaluate the capability of GSESNN for predicting potential indications for new drugs. Specifically, for each drug $r_i$, we remove all known drug-disease associations about the drug $r_i$ as a testing set and use all remaining associations as training samples. This setup ensures that information about the tested drug remains distinct from known drug-disease associations. For dataset selection, we utilize Gdataset, which is widely recognized as the gold standard dataset because it contains a comprehensive association collected by multiple data sources. In addition, for each drug, we select the nearest neighbor drug of a given drug in descending order based on drug similarity. Then we update its associations in the drug-disease graph with a part of its nearest neighbor drug’s association information. As shown in Fig. 3, we can find that GSESNN achieves the best results on both evaluation measures (AUROC of 0.8997 and AURPC of 0.3308), which demonstrates the superiority of GSESNN in predicting novel drug-disease associations.

### Parameter Analysis

**Fig. 4:**
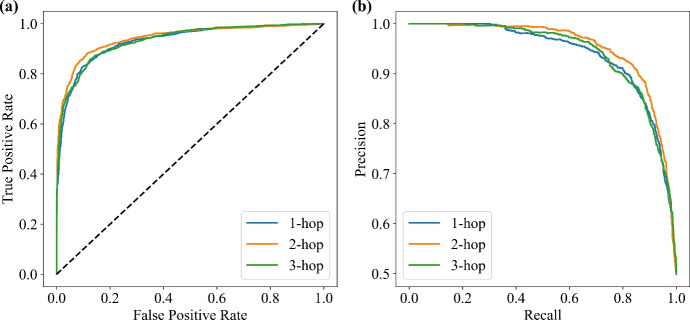
Impact of the number of hop on our model performance on Gdataset. **a** ROC curves of 10-fold cross validation results obtained by searching different hops. **b** Recall-precision curves of 10-fold cross validation results obtained by searching different hops

The effect of the depth of hop in subgraph extraction and the weight factor for the contribution of subgraph representation are experimentally verified. To investigate the effect of the hop depth when extracting subgraphs on model performance, we search the number of hop in the range of {1, 2, 3} and perform 10-fold cross validation on Gdataset. The mean results of AUROC and AUPRC are illustrated in Fig. 4. Obviously, extracting only 1 hop leads to the lowest performance, while 2 hop depth is able to endow the model with better performance. While continuing to increase the hop depth, the improvement is not noticeable. This suggests that the 2-hop information is sufficient to capture the necessary subgraph features for target node pairs. Therefore, we choose the hop as 2 in this paper. In order to verify the results of the effect of the weight factor on the model performance, we perform in {0.1, 0.2, ..., 0.9}, and perform 10-fold cross validation on Gdataset. The mean results of AUROC and AUPRC are shown in Table 3. With the increasing of the weight factor, the performance of the model continues to improve, and the performance of the model reaches the best when λ = 0.7. However, further increases in the weight factor lead to a decline in performance. Based on these findings, we select 0.7 as the optimal weight factor for this study.

**Table 3:** Impact of the weight factor value on our model performance on Gdataset

λ	AUROC	AUPRC
0.1	0.9393	0.9464
0.2	0.9447	0.9512
0.3	0.9450	0.9530
0.4	0.9497	0.9545
0.5	0.9451	0.9510
0.6	0.9444	0.9494
0.7	0.9499	0.9549
0.8	0.9472	0.9544
0.9	0.9465	0.9541

### Ablation Study

**Fig. 5:**
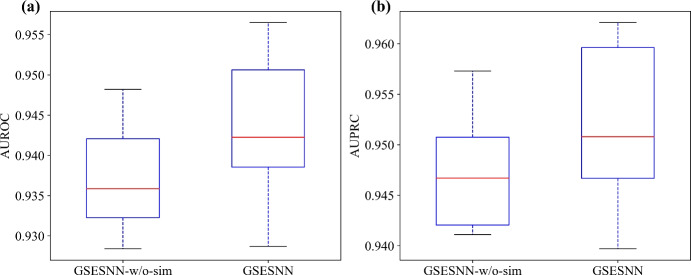
The results of whether or not the model utilizes similarity matrices

To verify whether our model heavily relies on drug and disease similarity matrices, we perform a 10-fold cross-validation on Gdataset with and without utilizing these matrices. We then perform a paired samples *t*-test on the AUROC and AUPRC values obtained from the experiment. The results are shown in Fig. 5: the average AUROC and AUPRC values for GSESNN-w/o-sim are 0.9369 and 0.9473, respectively, while those for GSESNN are 0.9439 and 0.9523. These results indicate minimal differences between the two experimental groups. The paired samples *t*-test yields a *t*-value of -2.1797 (*p* = 0.0572) for the AUROC data and a *t*-value of -1.7235 (*p* = 0.1189) for the AUPRC data. These results indicate that GSESNN does not heavily rely on drug and disease similarity matrices.

### Case Studies

To evaluate the practical applicability of GSESNN, we conduct case studies on two critical diseases: Alzheimer’s disease (AD) and Gastric Cancer (GC). AD is a progressive neurodegenerative disorder characterized by the insidious onset of dementia, which impairs memory, judgment, attention, and problem-solving skills, often leading to significant cognitive decline. Currently, no drugs are available that effectively treat AD, highlighting an urgent need for novel therapeutic options. Similarly, GC, a malignancy originating from the gastric mucosal epithelium, poses substantial treatment challenges. The current limited drug options for GC highlight the importance of expanding therapeutic alternatives to improve patient outcomes. By focusing on these two diseases in our case studies, we aim to evaluate GSESNN’s potential to identify promising drug candidates that address critical gaps in AD and GC treatment.

In our analysis, we use all known drug-disease associations in Gdataset as the training set and all missing drug-disease associations as the candidate set. After predicting probabilities for these candidate associations, we rank drug candidates by their predicted probability scores, identifying the top five drugs most likely to treat AD and GC. We then validate these predictions using well-known sources, including the Comparative Toxicogenomics Database (CTD) [23] and PubMed.^1^ Table 4 lists the drug candidates for which supporting evidence is obtained from these sources. As shown in the table, the top five ranked drugs based on predicted scores are corroborated by authoritative sources and literature, affirming the predictive accuracy of GSESNN. For example, Bleomycin [24] has been reported to inhibit the proliferation and migration of human GC cells by modulating the Smad signaling pathway through interaction with Smad3. Likewise, Phenobarbital [25], a medication traditionally used for epilepsy, has been found to regulate tau aggregation related to AD in a manner independent of neuronal activity. These results highlight GSESNN’s effectiveness in identifying viable drug candidates that align with current scientific findings.

**Fig. 6:**
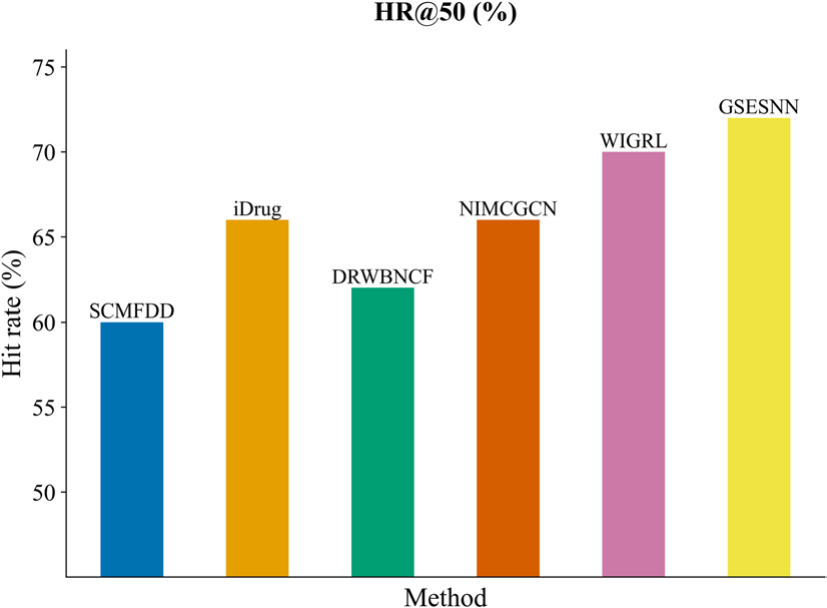
The hit rates of the top 50 drugs for treating Alzheimer’s disease predicted by our method and baseline

To validate the biological relevance of the predicted associations, we perform literature validation of the drugs with potential therapeutic potential for AD in the top 50 predictive scores of our model and compare them with all baseline models. The results, as shown in Fig. 6, show that our model has a hit rate of 72%, which is higher than all comparative models, demonstrating that our model is effective in identifying drugs with real therapeutic potential. For example: baclofen [26] significantly reduces amyloid-beta (Aβ)-induced mitochondrial cytochrome c release and caspase-3 activation in in vitro experiments in combination with acamprosate, thereby reducing neuronal apoptosis. In addition, Carbamazepine [27] improves cognitive function in AD mice by enhancing autophagy activity and reducing amyloid-beta42 levels in mouse modeling experiments.

**Table 4:** New candidate drugs ranked by GSESNN prediction scores for Alzheimer’s disease (OMIM:104300) and Gastric Cancer (OMIM:137215)

Diseases	Rank	DrugBank IDs	Candidate drugs	Evidences
AD	1	DB01174	Phenobarbital	[25]
	2	DB00747	Scopolamine	[28]
	3	DB00387	Procyclidine	[29]
	4	DB00413	Pramipexole	[30]
	5	DB00190	Carbidopa	[31]
GC	1	DB00541	Vincristine	[32]
	2	DB00515	Cisplatin	[33]
	3	DB00290	Bleomycin	[24]
	4	DB00958	Carboplatin	[34]
	5	DB00262	Carmustine	[35]

## Conclusion

In this paper, we propose GSESNN, a GCN-based method for drug repositioning that utilizes both subgraph information from the drug–disease association network and similarity feature information of drugs and diseases. Firstly, GSESNN extracts subgraphs for drug-disease pairs and utilizes GCN to learn subgraph representation. In addition, to distinguish between different drug-disease pairs with the identical subgraph topology, GSESNN extracts the global similarity feature representation of drug-disease node pairs. Specifically, GSESNN constructs similarity feature graphs based on the similarity matrices of drugs and diseases, and learns them by GCN to extract the embeddings of corresponding drugs and diseases. These embeddings are subsequently concatenated to form global similarity feature representation. By fusing these two learned representations and using a weight factor to balance their contributions, GSESNN obtains the final drug-disease association predictions. Extensive experimental results demonstrate that GSESNN achieves superior performance in drug repositioning tasks. Moreover, case studies illustrate GSESNN’s ability to predict previously unknown drug-disease associations for specific diseases.

Despite its effectiveness, GSESNN has some limitations. First, when known drug-disease pairs have limited samples, the model lacks sufficient feature information to fully support representation learning. Second, the model currently relies solely on drug-drug and disease-disease similarity data, without incorporating other biological data such as target information or drug-target interactions. Future work will aim to integrate additional biological knowledge to enhance the efficiency and reliability of model predictions.

## Footnotes

1 https://pubmed.ncbi.nlm.nih.gov.
